# First Real‐World Evidence of an AI‐Enhanced Digital Collaborative Care Model to Improve IBS Symptoms

**DOI:** 10.1111/nmo.70144

**Published:** 2025-09-04

**Authors:** Stephen E. Lupe, Joseph M. Olson, Kendra Kamp, Margaret Heitkemper, Mythili P. Pathipati, Madison L. Simons, Jordan Brown, Samuel N. Jactel, Miguel Regueiro, Anthony Lembo, Tiffany H. Taft

**Affiliations:** ^1^ The Cleveland Clinic Cleveland Ohio USA; ^2^ Ayble Health Boston Massachusetts USA; ^3^ University of Washington Seattle Washington USA; ^4^ Division of Gastroenterology Palo Alto Medical Foundation Palo Alto California USA; ^5^ The Rome Foundation Chapel Hill North Carolina USA

**Keywords:** brain–gut behavioral therapy, collaborative care model, dietary treatment, irritable bowel syndrome

## Abstract

**Background and Aims:**

Health systems struggle to deliver guideline‐recommended multidisciplinary care to patients with irritable bowel syndrome (IBS). Digital collaborative care models (DCCMs) that integrate technology with experienced providers offer a promising solution for improving IBS management. We aimed to evaluate whether a novel DCCM improved clinical outcomes in IBS.

**Methods:**

A prospective, longitudinal uncontrolled single‐arm study design was used to assess the Ayble Health program. Participants were recruited online via social media, clinic, or employer. Data were prospectively collected from 202 participants (78% female; 78% white) with active IBS symptoms at baseline (≥ 75 on the IBS symptom severity scale (IBS‐SSS)) and completed at least one follow‐up symptom survey. All participants engaged in at least one care pathway: (1) a multidisciplinary care team, (2) a nutrition program with a personalized elimination diet, and (3) a brain–gut behavioral therapy (BGBT) program. Each pathway was supported by AI algorithms trained on a large, multimodal GI dataset to identify and communicate key trends in patient‐reported outcomes, further personalizing care plans.

**Results:**

Of the 202 participants, 197 (98%) participated in the nutrition pathway, 152 (75%) the BGBT pathway, and 156 (77%) the care team pathway. The majority of participants (62%) enrolled in all three pathways. Participants experienced a 140‐point decrease in IBS‐SSS, on average, with 86% experiencing a ≥ 50‐point reduction.

**Conclusion:**

The novel DCCM successfully delivered evidence‐based care to participants with active IBS symptoms, with clinically significant, sustained symptom relief. Randomized clinical trials are recommended to assess cost and treatment efficacy compared to standard of care approaches.

## Graphical abstract

Participants engaged in the DCCM platform (Ayble Health) and self‐selected a care pathway. Seventy‐five percent of participants who engaged with a novel DCCM using a combination of brain–gut behavior therapy, dietary modification, and/or clinical care teams, enhanced by AI, experienced clinically significant symptom relief sustained up to 42 weeks.

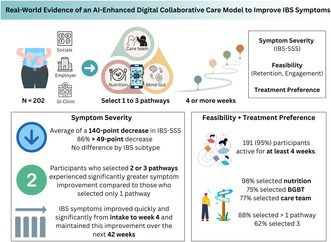

**Summary**

- Background
  ○ Access to adjunctive diet and brain–gut behavioral therapies for IBS remains a significant clinical challenge. Digitally enabled collaborative care models (DCCMs) enhanced by artificial intelligence (AI) may offer a viable solution.
- Findings
  ○ Seventy‐five percent (173 out of 202) of IBS participants who engaged with a novel DCCM (Ayble Health) using a combination of brain–gut behavior therapy, dietary modification, and/or clinical care teams, enhanced by AI, experienced clinically significant symptom relief sustained up to 42 weeks.
- Implications for patient care
  ○ DCCMs enhanced by AI have meaningful positive impacts on IBS symptoms and could mitigate ongoing barriers to accessing adjunctive treatments for IBS, allowing integrated care to scale.

## 1 Introduction

Irritable bowel syndrome (IBS) remains a clinically challenging condition affecting around 10% of the adult global population [1]. Despite advances in our understanding of IBS's complex physiological mechanisms, including diet, eating behavior, stress, and the brain–gut‐microbiome axis [2], many patients continue to be inadequately managed. The individual and societal impacts of IBS are extensive [2], with 2024 annual healthcare expenditures estimated at $13,000 per patient. Current clinical practice guidelines recommend multidisciplinary treatments for IBS, including dietary modifications and brain–gut behavioral therapies (BGBTs), which have shown robust efficacy data [3, 4, 5, 6].

Unfortunately, the multidisciplinary structures shown to produce superior IBS patient outcomes in research [7] are rarely available in real‐world clinical practice. For example, while over 80% of patients report a link between IBS symptoms and their diet [6], a 2022 survey found that over half of gastroenterologists do not provide dietary guidance [8] (91% would refer to a dietitian, 42% of gastroenterologists lacked access) [8]. Additionally, BGBTs [3] like gut‐directed hypnosis (GDH) and cognitive–behavioral therapy (CBT) are highly effective in reducing symptoms [3] and reducing healthcare utilization [9] while increasing patient satisfaction. However, in a 2023 survey, 92% of gastroenterologists reported a shortage of behavioral health providers, and 87% stated these providers' limited availability was a barrier to using BGBTs [10, 11].

Leveraging technology is a promising solution to increase access to multidisciplinary IBS care, especially considering the substantial shift to telehealth since the COVID‐19 pandemic [12]. However, telehealth alone does not simplify navigating the healthcare system, which many patients find burdensome and stressful [13]. The fragmentation of multidisciplinary care across multiple clinics often leads to duplicative efforts that hinder access to effective care, and virtual GI clinics have struggled to ameliorate this siloed delivery of limited resources. Digital solutions such as smartphone apps [14, 15, 16] are currently limited in their capacity to meet the needs of most IBS patients, can have high attrition, and are not easily coordinated with local providers.

Collaborative care models (CCM) that use a multiprofessional approach, including physicians, nurses, behavioral health, and dietitians, to deliver structured patient care plans are well‐studied in primary care [17, 18]. In gastroenterology, the inflammatory bowel disease (IBD) medical home CCM has demonstrated significant value in improving patient outcomes and reducing cost [19]. Unfortunately, similar models for IBS are limited due to systemic barriers, most often being provider time, cost, and a lack of qualified clinicians. In short, while multidisciplinary care is critical to successful IBS treatment, it is untenable to expect every gastroenterology clinic to implement their own CCM. Novel solutions are needed to facilitate scalable implementation of collaborative care for IBS, where gastroenterology providers have access to experienced multidisciplinary teams that serve as invested partners in their patients' care.

Digital collaborative care models (DCCMs) that integrate technology with expert providers are a potential next step in the evolution of IBS management. In particular, clinician‐supervised artificial intelligence (AI) tools could facilitate and enhance the precision of individualized IBS treatment, potentially reducing trial‐and‐error approaches. Ayble Health is a new DCCM designed to deliver scalable and precise dietary and BGBT care for individuals with IBS. Our primary aim was to evaluate the improvement in IBS symptoms across IBS subtypes for participants using Ayble Health. Secondary aims include identifying differences in response across baseline symptom severity subgroups, treatment preferences, and demographic characteristics (sex at birth, ethnicity). We also aimed to evaluate the stability of treatment response over time.

## 2 Methods

### 2.1 Design

**FIGURE 1:**
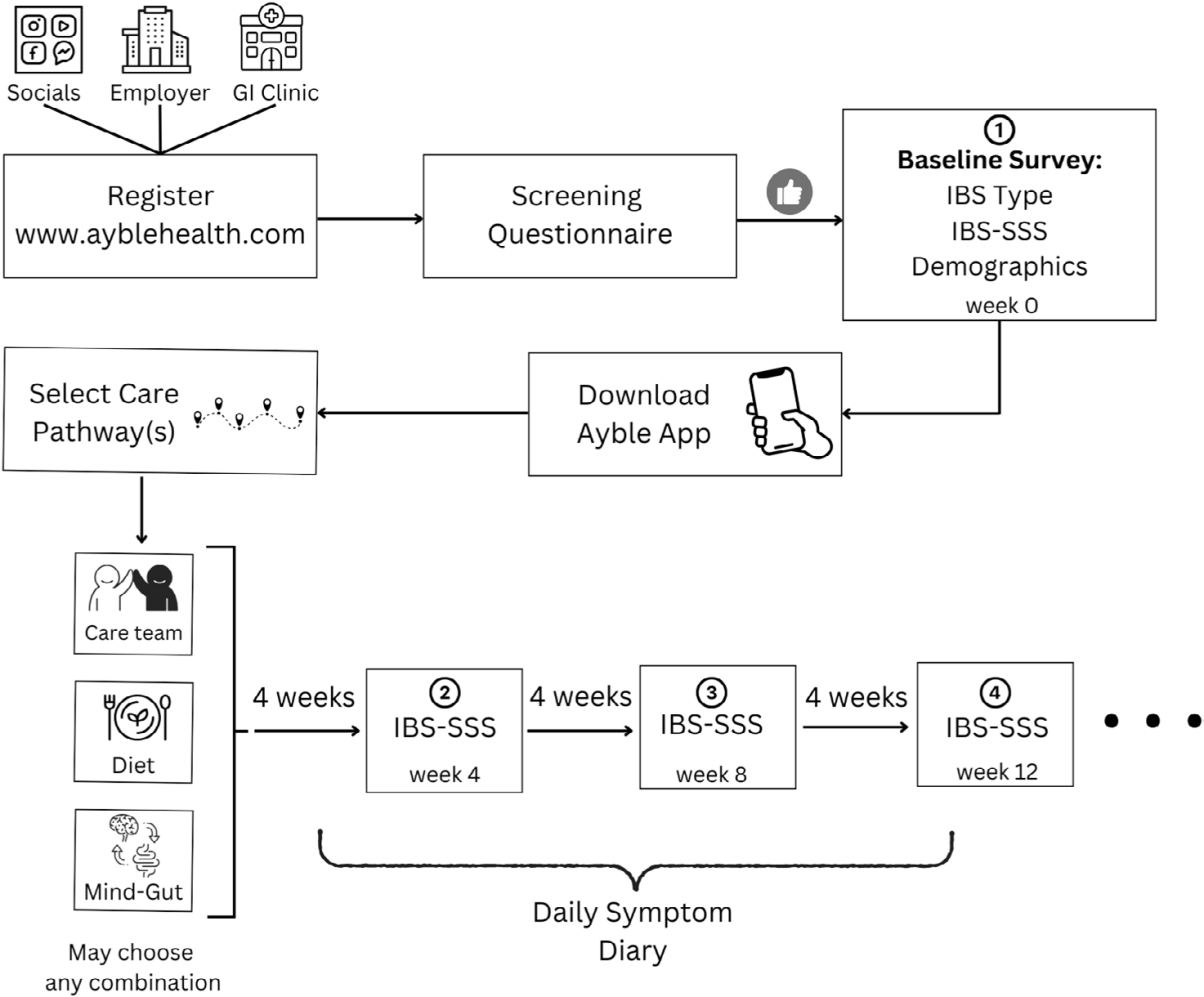
Study design.

A prospective, longitudinal single‐arm study design was used to assess the Ayble Health program (see Figure 1). Participants were enrolled in three ways: (1) self‐referred, (2) referred via GI provider or clinic, and (3) referred via employer benefits program. To be included in the analysis, participants had to consent to Ayble Health's End User License Agreement (EULA), which was provided to them digitally and included consent to have anonymized data collected and used for research purposes.

### 2.2 Participants

Participants included those who registered for the Ayble Health program via a website (www.ayblehealth.com) which was advertised via social media, their employer, or in outpatient gastroenterology practices (Figure 1). Participants were included if they self‐reported a prior diagnosis of IBS and had active IBS symptoms at intake (≥ 75 on IBS‐SSS). Participants were excluded from the study if they had a prior diagnosis of other GI conditions (e.g., IBD, celiac disease, and gastroesophageal reflux disease), pregnancy, a history of an eating disorder (e.g., bulimia and anorexia), and/or a history of bowel surgery, or if their IBS symptoms were in remission at intake.

### 2.3 Procedure

Following exclusions, participants downloaded a supportive smartphone application (iPhone or Android) to access program content and log follow‐up assessments.

#### 2.3.1 Care Model Description

**FIGURE 2:**
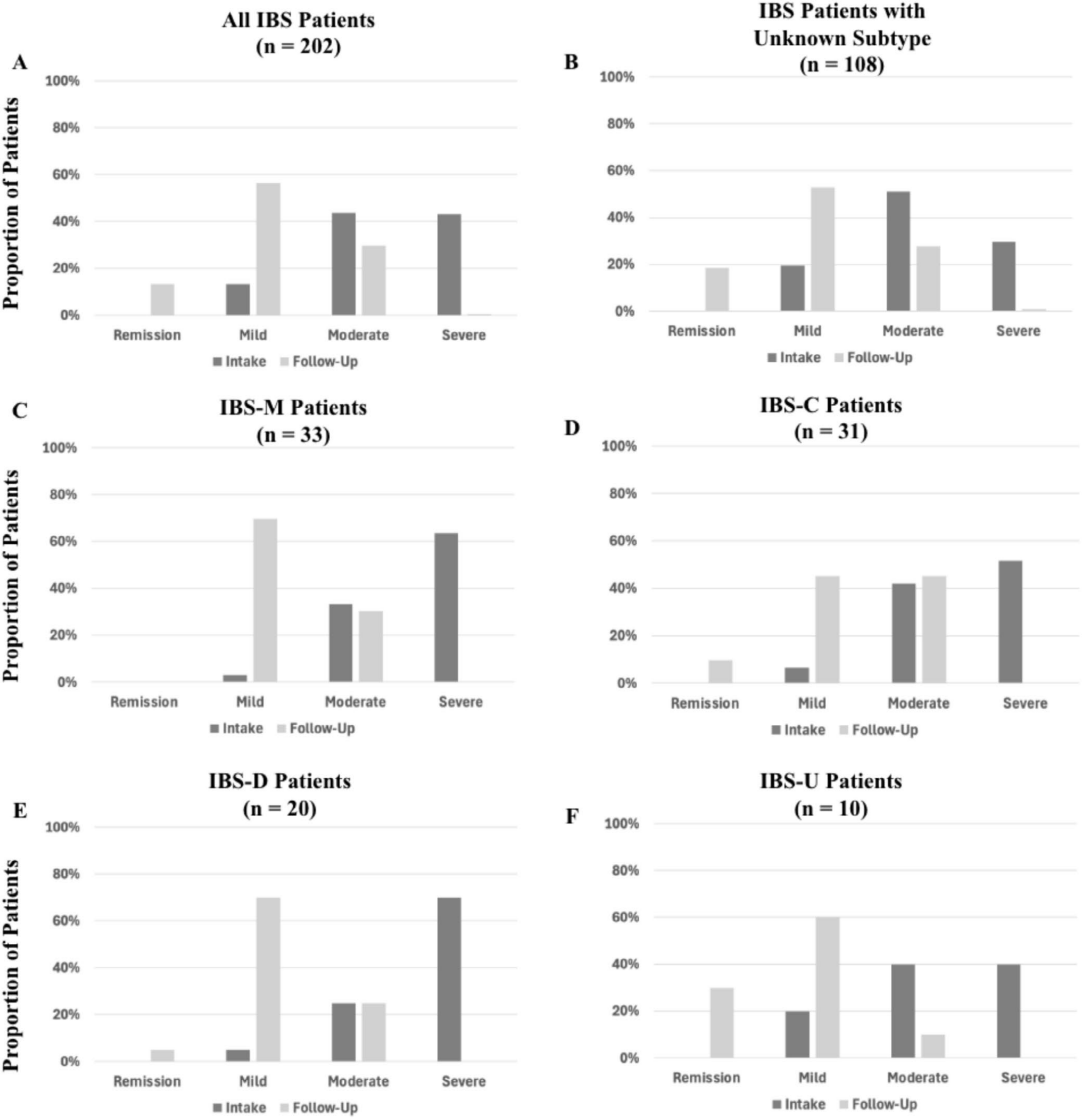
IBS‐SSS symptom severity at intake vs. follow‐up across IBS subtypes. “Follow‐up” is the average IBS‐SSS score derived from all follow‐up surveys provided per patient.

The Ayble Health DCCM comprises three core care pathways that participants could interact with over the course of their program. These pathways included (1) a care team, (2) a nutrition program, and (3) a BGBT program. Participants were allowed to engage in any one or combination of the three pathways and were given the choice of which pathway(s) they enrolled in; this was not based on symptom severity or presentation at baseline (Figure 2).

##### 2.3.1.1 Care Team

The care team primarily included National Board Certified Health and Wellness Coaches (NBC‐HWC) overseen by a GI psychologist, dietitian, and gastroenterologist, and offered motivational interviewing, goal setting, support, and guidance throughout the Ayble Health program. Patients could access their care teams via periodic synchronous virtual visits and asynchronous text messaging. The care team was assisted by AI, which was designed to summarize and report on patient health outcomes and engagement over the course of their program, and predict possible next steps in care delivery [20]. The care team had flexibility to refer participants to certain care pathways or components within a pathway based on AI feedback in order to further personalize the participants' treatment.

##### 2.3.1.2 Nutrition Program

The nutrition program included a 4–10+ weeks personalized elimination diet. The Ayble Health elimination diet has four “phases” (Identification, Elimination, Reintroduction, and Application) supervised by the care team to ensure reintroduction was properly implemented. The phases are detailed extensively in a previous report [20], and are briefly described next. In the Identification phase, participants ate normally for 3 weeks while tracking their symptoms and diet daily. At the end of this phase, a proprietary machine learning algorithm trained on the GI literature, previous patient data, and the patient's personal symptoms and diet, suggested up to five potential “suspect” trigger food categories (e.g., Alliums) for the patient's consideration. In the Elimination phase, participants eliminated or reduced their intake of up to five suspected trigger food categories while continuing to track their symptoms. In the Reintroduction phase, participants reintroduced each trigger food category one at a time in a “food challenge” Participants could challenge as many or as few food categories as were relevant or interesting to them. Participants were also allowed to test multiple food items within a category (e.g., test both garlic and onions within the Alliums category). When the patient finished their food challenges, they entered the Application phase, where the insights from the previous three phases were applied to their new, modified diet.

##### 2.3.1.3 BGBT Program

The BGBT program was a 4–10+ weeks curriculum of daily guided GDH, acceptance and commitment therapy (ACT), and cognitive behavioral therapy (CBT) audio recordings and texts based on the established literature applying these concepts to IBS [3], including the University of Washington Comprehensive Self‐Management Intervention for IBS [21]. A new hypnotherapy protocol was created by the study psychologists (TT, SL) who have extensive experience using GDH. Content was drawn from multiple sources to best align with standard GDH protocols while also respecting copyright. Content was listed in a specific order based on study psychologist consensus. Subsequent material was “unlocked” contingent upon completion of BGBT activities from previous days. There were no time constraints regarding when subsequent content was unlocked. Over the course of the program, participants completed psychoeducational readings, GDH for bowel symptoms, journal prompts, diaphragmatic (“deep”) breathing, and muscle relaxation exercises. ACT and CBT activities were approximately 1–5 min in duration, while GDH activities were each 15–20 min. Approximately 20 h of unique BGBT content was available.

### 2.4 Measures

#### 2.4.1 Measures

##### 2.4.1.1 Demographic and Clinical Information

Participants completed a baseline intake questionnaire, including demographic information (sex at birth, race/ethnicity).

##### 2.4.1.2 IBS Symptom Severity

IBS symptom severity using the IBS‐SSS [22]. Scores ranged from 0 to 500 with higher scores indicating more severe IBS symptoms. Symptoms were evaluated on a 10‐point visual analog scale (VAS) for frequency and severity. The IBS‐SSS asked about abdominal pain (severity and number of days with pain), bloating and flatulence, satisfaction with bowel habits, and symptom interference. Baseline IBS‐SSS was a 10‐day retrospective measure. After enrollment, participants completed daily symptoms via a smartphone companion app‐based on an adapted version of the IBS‐SSS [20].

##### 2.4.1.3 Feasibility and Treatment Preference

We also collected data on the proportion of participants who engaged in the Ayble Health program for at least 4 weeks as well as which care pathway or combination of care pathways the participants selected.

#### 2.4.2 Statistical Analysis

All data were analyzed using JASP (version 0.18.3 Apple Silicon for MacOS [23]) and Python (version 3.9.13). A *p* value of 0.05 was considered statistically significant. Descriptive statistics were reported as means (M), counts, or percentages. Follow‐up survey data were aggregated from daily symptoms into 4‐week long time periods (i.e., Weeks 1–4, Weeks 5–8, etc.). Changes in IBS symptom scores between intake and follow‐up points (i.e., Weeks 4, 8, 12, and 13+) were analyzed per cohort (IBS subtype, treatment preference, intake symptom severity, sex at birth, ethnicity) using mixed methods ANOVAs. The ANOVAs were decomposed using Bonferroni‐corrected independent or paired *t*‐tests where applicable. For simplicity in interpretation, the *p*‐values themselves were corrected by dividing by the number of comparisons performed. Partial eta squared (*η*_p_^2^) was used to determine ANOVA effect size (*η*_p_^2^ = 0.01 small, *η*_p_^2^ = 0.06 medium, *η*_p_^2^ = 0.14 large [24]).

All 202 participants provided at least one follow‐up survey (M = 19.25 surveys, SD = 22.70). When multiple follow‐up surveys were logged, they were aggregated into one follow‐up score per patient to provide a conservative description of the participants' overall care during the program.

## 3 Results

### 3.1 Demographic and Clinical Characteristics

Data were prospectively collected from 202 participants (78% female; 78% white; age M = 41.53, SD = 12.28; see Table 1) who self‐reported active IBS symptoms (≥ 75 on the IBS‐SSS). Ninety‐four participants (46.53%) self‐reported an IBS subtype (i.e., IBS‐Diarrhea, *n* = 20; IBS‐Constipation, *n* = 31; IBS‐Mixed, *n* = 33; IBS‐Unspecified, *n* = 10), and 108 (53.47%) reported IBS with an unknown subtype.

**TABLE 1:** Clinical characteristics.

Patient cohort	Sample size	Percent (%)
All patients	*n* = 202	100.00
Age (years)	41.53 ± 12.28	
Range	19.36–69.60	
IBS subtype		
IBS‐C	*n* = 31	15.35
IBS‐D	*n* = 20	9.90
IBS‐M	*n* = 33	16.34
IBS‐U	*n* = 10	4.95
IBS unknown subtype	*n* = 108	53.47
Treatment preference		
Care team	*n* = 0	0.00
BGBT	*n* = 1	0.50
Nutrition	*n* = 24	11.88
Care team + BGBT	*n* = 4	1.98
Care team + nutrition	*n* = 26	12.87
Nutrition + BGBT	*n* = 21	10.40
Care team + nutrition + BGBT	*n* = 126	62.38
Symptom severity at intake		
Mild	*n* = 27	13.37
Moderate	*n* = 88	43.56
Severe	*n* = 87	43.07
Sex at birth		
Male patients	*n* = 43	21.29
Female patients	*n* = 159	78.71
Ethnicity		
Asian	*n* = 16	7.92
White	*n* = 158	78.22
Black or African American	*n* = 5	2.48
Hispanic or Latino	*n* = 13	6.44
All other	*n* = 10	9.41

### 3.2 Treatment Preference & Feasibility

Out of 202 IBS participants, 197 (98%) selected the nutrition pathway, 152 (75%) selected the BGBT pathway, and 156 (77%) selected the care team pathway. The majority of participants (177 or 88%) selected more than one pathway, with 126 (62%) selecting all three. The participants who selected only one pathway almost exclusively selected nutrition (24/25 96%). Retention and engagement were high, with 191 (95%) participants active for at least 4 weeks.

### 3.3 IBS Symptom Improvement

Participants demonstrated a 140‐point decrease in IBS‐SSS (*p* < 0.001, *η*_p_^2^ = 0.74), on average, with the majority of participants (173/202 or 86%) achieving clinically significant IBS symptom amelioration from intake to follow up (≥ 50 on the IBS‐SSS). A detailed analysis across treatment preference, IBS subtypes, intake symptom severity, sex at birth, and ethnicity is presented in Table 2.

**TABLE 2:** Analysis of symptom improvement by cohort.

Patient cohort	Average symptom change	*p*	Clinical significance	Sample size
All patients	−139.97*	< 0.001	85.64%	*n* = 202
IBS subtype				
IBS‐C	−158.83*	< 0.001	90.32%	*n* = 31
IBS‐D	−179.28*	< 0.001	90.00%	*n* = 20
IBS‐M	−172.47*	< 0.001	96.97%	*n* = 33
IBS‐U	−178.38*	< 0.001	100.00%	*n* = 10
IBS unknown subtype	−116.00*	< 0.001	78.70%	*n* = 108
Treatment preference				
Care team	na	na	na	*n* = 0
BGBT	0.00	na	na	*n* = 1
Nutrition	−95.50*	< 0.001	75.00%	*n* = 24
Care team + BGBT	−136.67*	0.952	75.00%	*n* = 4
Care team + nutrition	−123.64*	< 0.001	80.77%	*n* = 26
Nutrition + BGBT	−153.79*	< 0.001	95.24%	*n* = 21
Care team + nutrition + BGBT	−150.24*	< 0.001	87.30%	*n* = 126
Symptom severity at intake				
Mild	−46.50	0.009	51.85%	*n* = 27
Moderate	−111.47*	< 0.001	85.23%	*n* = 88
Severe	−197.79*	< 0.001	96.55%	*n* = 87
Sex at birth				
Male patients	−133.93*	< 0.001	81.40%	*n* = 43
Female patients	−141.60*	< 0.001	86.79%	*n* = 159
Ethnicity				
Asian	−104.39*	0.008	56.25%	*n* = 16
White	−145.52*	< 0.001	87.98%	*n* = 158
Black or African American	−135.19*	0.010	100.00%	*n* = 5
Hispanic or Latino	−128.30*	0.002	84.62%	*n* = 13
All other	−126.65*	0.002	90.00%	*n* = 10

*Note:* *p* values are Bonferroni‐corrected for a family of 23. * = clinically significant reduction of 50 points on the IBS‐SSS.

#### 3.3.1 Symptom Improvement Across IBS Subtypes

**FIGURE 3:**
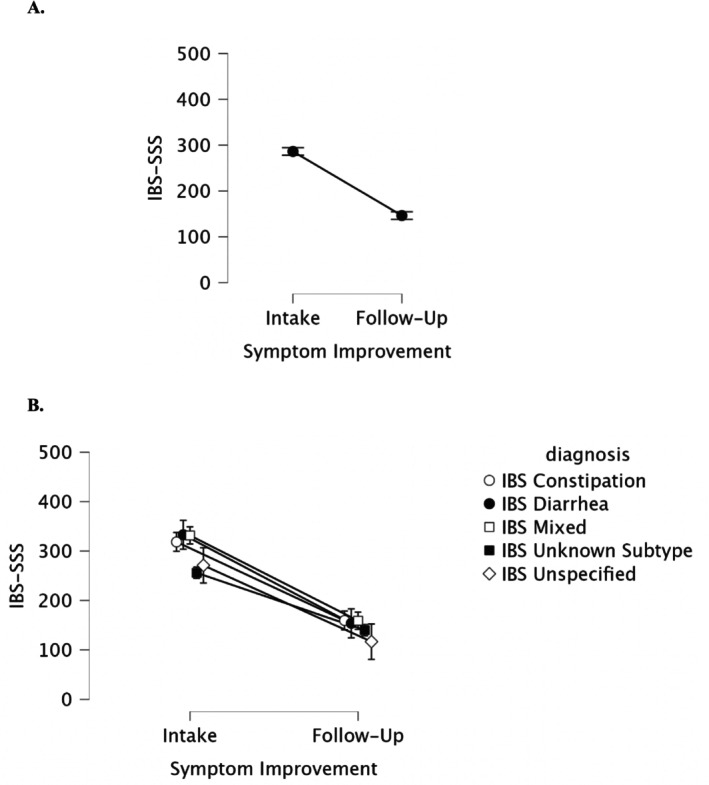
IBS‐SSS score symptom improvement. (A) Main effect of symptom improvement. (B) Interaction between symptom improvement and subtype. Error bars are 95% confidence intervals.

Figure 3 displays intake vs. follow‐up symptom severity as measured by the IBS‐SSS (remission 0–74, mild 75–175, moderate 176–300, and severe > 300), per subtype. Participants experienced substantial improvement in their IBS symptoms regardless of subtype (*p* < 0.001, intake M = 286.24, SD = 88.38; follow‐up M = 146.27, SD = 62.28), see Figure 3A. Participants with an unknown IBS subtype had lower symptoms compared to IBS C, D, and M, at intake (Bonferroni‐corrected *ps* < 0.01) but not at follow‐up (Bonferroni *ps* > 0.9), see Figure 3B.

#### 3.3.2 Symptom Improvement Across Treatment Preference

Participants who selected two or more pathways experienced significantly greater symptom improvement (M = −141.08, SD = 78.86) compared to those who selected only one pathway (M = −77.75, SD = 76.60, *p* = 0.003). An improvement was also observed for rates of clinically significant improvement for multiple pathways (154/177 or 87%) vs. one pathway (19/25 or 76%), but the difference was not significant (*p* = 0.14).

#### 3.3.3 Symptom Improvement Across Intake Symptom Severity, Sex at Birth and Ethnicity Cohorts

Participants with severe symptoms at intake experienced greater symptom reduction (M = −197.79, SD = 68.38) compared to participants whose symptoms were moderate (M = −111.47, SD = 60.33) or mild (M = −46.50, SD = 59.56). Participants with moderate symptoms at intake improved more than participants with mild symptoms (Bonferroni *ps* < 0.001). No differences in symptom improvement were observed across sex at birth or ethnicities (Bonferroni *ps* > 0.63), suggesting that each cohort experienced similar symptom improvement.

#### 3.3.4 Stability of Symptom Improvement Over Time

**FIGURE 4:**
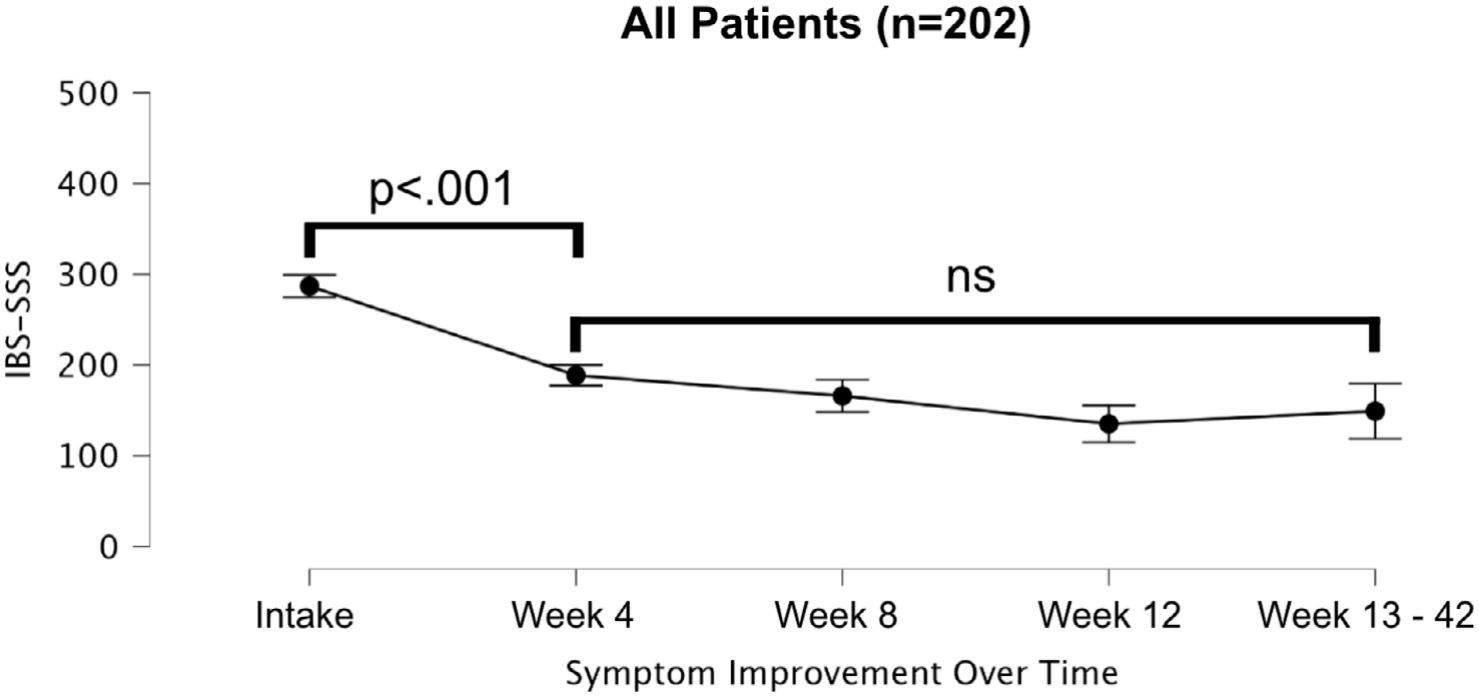
IBS‐SSS symptom stability. Post hoc comparisons are Bonferroni‐corrected. ns = *p* > 0.05. Error bars are 95% confidence intervals.

Participants' IBS symptoms improved quickly and significantly from intake (M = 288.20, SD = 87.64) to week 4 (*p* < 0.001, M = 188.68, SD = 78.88) and maintained this improvement over the next 42 weeks (*p* < 0.001, M = 149.22, SD = 92.29) (see Figure 4).

## 4 Discussion

Multidisciplinary care under a collaborative model is the ideal treatment paradigm for patients with IBS in terms of favorable outcomes and lower costs. However, widespread implementation of this model remains a challenge due to meaningful drawbacks associated with both traditional clinics and unsupported mobile app‐based modalities. An approach that is scalable, accessible, and fosters engagement with both patients and their treating providers is a promising and necessary step in the evolution of multidisciplinary gastroenterology practice. Preliminary results from a novel DCCM (Ayble Health) targeting both dietary modification and BGBT alongside a care team demonstrated significant symptom improvements, with an average IBS‐SSS reduction of 140 points (46%) and 86% of participants reporting clinically significant symptom relief (≥ 50 on the IBS‐SSS). Participants, regardless of IBS subtype, had a rapid response to the program within 4 weeks that was sustained through 42 weeks.

There are several potential mechanisms to explain these positive outcomes. First, unlike standalone apps, Ayble Health utilizes a collaborative multidisciplinary care team, which includes health coaches under medical supervision, coordinated with local gastroenterologists to enhance patient engagement with the program material. Health coaching emphasizes a patient‐centered collaborative approach that guides individuals toward realistic goals [25]. When combined with standard medical care, health coaching increases adherence to healthy behaviors such as exercise, dietary modification, and medication adherence [26]. Almost three‐quarters of participants in this study chose to use the care team pathway, which suggests the human element is critical for achieving positive outcomes. The combination of comprehensive self‐directed material with both synchronous and asynchronous access to a health coach is a plausible explanation for this study's findings, which should be investigated in future controlled trials.

A second potential mechanism is the coordinated and simultaneous application of two widely accepted, efficacious treatments for IBS: dietary modification and BGBTs. Consistent, robust outcomes exist for exclusion diets and BGBTs, with 60%–80% of IBS patients showing clinically significant reductions in symptoms as measured by at least a 50‐point reduction in the IBS‐SSS [22]. Prior RCTs in this space are limited to evaluating either a dietary intervention or a BGBT, leaving our understanding of their concurrent application limited to anecdotal clinical evidence. The reality is there is considerable overlap between diet and psychosocial factors in the IBS patient experience that influence outcomes. The present study is the first to assess the potential benefits of delivering both dietary and BGBT approaches together. Our results demonstrate similar efficacy to those found in prior research; future RCTs should aim to evaluate the comparative effectiveness of a combined approach delivered via a DCCM versus traditional siloed BGBT or dietary treatment models.

A third potential mechanism behind these results is the interplay between the challenges of dietary elimination and the skills gained from BGBTs [27]. A proportion of patients using elimination diets report impairments to quality of life, especially as the number of eliminated foods increases. The BGBT content of a DCCM could afford patients with concurrent skills to mitigate stress or anxiety associated with eliminating and reintroducing foods, in addition to the typical BGBT targets of managing gut symptoms.

The findings in this study underscore the need to expand multidisciplinary care for IBS. As of 2024, almost all multidisciplinary GI clinics in the US exist in academic medical centers. In the U.S., there are approximately 450 vetted mental health clinicians as listed in the Rome Foundation Psychogastroenterology directory (https://romegipsych.org/) who are vetted to deliver care to millions of patients with chronic digestive illness (as of November 2024) [28]. Listings for low‐FODMAP trained GI dietitians are similar, ranging from 81 on the International Foundation for Gastrointestinal Disorders (https://iffgd.org/resources/dietitian‐listing/) to around 150 (https://www.katescarlata.com/fodmapdietitians). It is untenable to expect every gastroenterology clinic to implement its own CCM, but DCCMs like Ayble Health may provide a scalable solution to bridge this gap. This would enable offering evidence‐based interventions to a greater number of patients regardless of geographic location.

### 4.1 Limitations and Future Directions

This study has some limitations. We relied on patient self‐reported diagnosis of IBS and therefore did not have confirmation using Rome IV diagnostic criteria, nor did we document any comorbid upper DGBI such as functional dyspepsia. However, baseline IBS‐SSS scores allowed us to benchmark symptom severity. We did not evaluate disease‐related quality of life, which is an important outcome for future studies. The absence of a randomized controlled design (e.g., with treatment‐as‐usual group) increases the risk for bias and placebo effects. We utilized conservative statistical analyses to attempt to reduce bias, and the change in IBS‐SSS score is large enough to suggest the findings extend beyond typical placebo effect rates seen in IBS treatment trials. The majority of participants were self‐referred and motivated to sign up for the Ayble Health program, suggesting they were interested in the treatment modalities and had access to the requisite smartphone. Access to the technology needed to use a DCCM may be a self‐selecting variable that contributes to the success of the program. The ability to afford a smartphone and the technology literacy to use it may differ across socioeconomic status and education backgrounds, making generalization to large populations difficult. Future studies should attempt to control for these variables.

## Author Contributions

**Stephen E. Lupe:** conceptualization (lead); writing – original draft (equal); writing – review and editing (lead); supervision (equal). **Joseph M. Olson:** conceptualization (equal); methodology (equal); investigation (equal); writing – review and editing (equal); supervision (equal). **Kendra Kamp:** conceptualization (equal); methodology (equal); investigation (equal); writing – review and editing (equal); supervision (supporting). **Margaret Heitkemper:** conceptualization (supporting); methodology (supporting); investigation (equal); writing – review and editing (equal). **Mythili P. Pathipati:** conceptualization (supporting); methodology (supporting); investigation (supporting); writing – review and editing (supporting). **Madison L. Simons:** conceptualization (equal); methodology (equal); investigation (supporting); writing – review and editing (equal). **Jordan Brown:** conceptualization (equal); methodology (equal); investigation (equal); writing – review and editing (supporting). **Samuel N. Jactel:** conceptualization (equal); methodology (equal); investigation (equal); writing – review and editing (equal); supervision (equal). **Miguel Regueiro:** conceptualization (supporting); methodology (supporting); writing – review and editing (equal). **Anthony Lembo:** conceptualization (supporting); methodology (supporting); writing – review and editing (equal). **Tiffany H. Taft:** conceptualization (co‐lead); methodology (equal); investigation (equal); writing – review and editing (equal); supervision (equal).

## Disclosure

Stephen E. Lupe: Scientific Advisor, Boomerang Health; Paid Lecturer, Takeda Pharmaceuticals; Scientific Advisor; Healthnix. Joseph M. Olson: employee at Ayble Health Inc. with stock options. Mythili P. Pathipati: consultant at Ayble Health Inc. Samuel N. Jactel: officer at Ayble Health Inc. with stock options. Tiffany H. Taft: Scientific Advisor, Ayble Heath Inc.; ownership interest, Oak Park Behavioral Medicine LLC. Anthony Lembo: GSK, Takeda, Ardelyx, Vibrant, Atmo, BioAmerica, Ironwood, J&J, Bristol Myer Squibb, Allurion. Miguel Regueiro: Advisory Boards and Consultant (both) for Abbvie, Johnson and Johnson, UCB, Takeda, Pfizer, BMS, Organon, Amgen, Genentech, Gilead, Salix, Prometheus, Lilly, Celgene, Boehringer Ingelheim Pharmaceuticals Inc. (BIPI), Celltrion, Roche, Merck, Sanofi, Biocon, Abavax.

## Conflicts of Interest

The authors declare no conflicts of interest.

## Data Availability

The data that support the findings of this study are available on request from the corresponding author. The data are not publicly available due to privacy or ethical restrictions.
